# A preliminary study on analysis of lower limb energy during walking in the patients with knee replacement

**DOI:** 10.1016/j.heliyon.2024.e27960

**Published:** 2024-03-13

**Authors:** Haifei Zhou, Yuying Zhang, Archit Agarwal, Graham Arnold, Weijie Wang

**Affiliations:** a University Department of Orthopaedic and Trauma Surgery, Ninewells Hospital and Medical School, University of Dundee, Dundee, DD1 9SY, United Kingdom; b Department of Orthopedics, S P Wahi ONGC Hospital, Dehradun, 248003, India

**Keywords:** Total knee arthroplasty, Unicondylar knee arthroplasty, Gait analysis, Mechanical energy, Recovery coefficient

## Abstract

**Background:**

Knee replacement surgeries are used to reduce pain and enhance functionality for individuals with knee arthritis. It is predicted that the annual volume of total knee replacement surgeries conducted in the US will surge by a substantial 673% by 2030. Though a lot of studies have done gait analysis on patients with knee replacement, little research is on energy changes in the lower limbs during gait. This study aimed to investigate the mechanical energy changes in the lower limbs for patients with total knee arthroplasty (TKA) and unicondylar knee arthroplasty (UKA), and ultimately to provide a specific tool to analyze limb energy during gait in clinical practice.

**Methods:**

10 TKA and 8 UKA patients were recruited for gait analysis. The control group consisted of 11 individuals without knee replacement surgery. Vicon motion capture system and Plug-in-Gait model were used to collect gait data to obtain marker coordinates and gait parameters. The kinetic energy, potential energy, and rotational energy for each segment in the lower limbs were calculated. The energies in the centre of pelvis were considered as the approximate to the centre of mass. The energy recovery coefficients were analysed for each segment during gait. SPSS was used to identify the differences between different groups.

**Results:**

The results showed that during walking, the upper leg had the highest recovery coefficient, approximately 40%, followed by the foot at 10%, and the lowest recovery coefficient was observed in the lower leg, approximately 1–3%. However, the energy recovery coefficients at the centre of pelvis were significantly higher in the control group than the TKA and UKA groups by roughly 12%–15%.

**Conclusions:**

The energy difference between the operative and non-operative sides is not significant regardless of the type of surgery. The TKA and UKA groups were more active in potential energy than control group. The upper leg has the highest recovery efficiency of kinetic and potential energy exchanges when walking. The control group used the energy for whole body is better than the patient groups. This study provides a new and useful way to analyze mechanical energy in the lower limbs during gait and could be applied in clinical practice.

## 1 Introduction

Arthritis is a group of musculoskeletal disorders and also one of the main causes of disability in the elderly population [1]. Osteoarthritis (OA) is the most common type of arthritis, and is characterized by joint degeneration, particularly in the knee, and involvement of multiple joints. OA affected 344 million people worldwide in 2019 [2]. The main symptoms of OA are increased pain and decreased function. Total knee arthroplasty (TKA) is a popular procedure, and OA is the main surgical indication for TKA [3]. TKA is a high-efficiency method to improve patient's quality of life and the level of limb function for people who got OA. The unicondylar knee arthroplasty (UKA) is an increasingly popular alternative to TKA for the treatment of OA. UKA offers OA patients proprioception, superior functional outcomes [4], and less intraoperative blood loss compared to TKA [5].

As the number of TKA and UKA procedures has increased, many studies have demonstrated the efficacy of TKA and UKA in patients with OA, but there are still many post-operative recovery problems gradually exposed. Andrew and Jennifer indicated the postoperative walking speed of patients decreased about 18% when comparing with healthy adults [6]. A study reported that postoperatively, gait assessments reveal significant improvements in stance time, stride length, and other aspects for TKA and UKA patients [7]. The total range of knee motion during walking is lower in TKA patients than in healthy people, while TKA patients have reduced balance and are prone to falls [[8], [9], [10], [11]]. Knee arthroplasty patients were able to produce the same knee extension moments during walking as healthy control limbs [12], but the TKA group reported reduced functional mobility, increased temporal variability of stride length, and impaired balance [13]. Several studies have noted that patients after TKA and UKA have greater difficulty with kneeling, squatting, lateral movements, turning, cutting, weight bearing, lower limb strengthening, tennis, dancing, gardening and sexual activity compared to healthy adults [14]. These studies compared post-joint replacement patients with healthy adults from various aspects to gain a comprehensive understanding of the recovery of post-joint replacement patients. However, most of these studies predominantly focus on spatiotemporal parameters, gait kinetics, or knee joint functionality [[6], [7], [8], [9], [10],^12^,^13^]. Nonetheless, there is relatively limited research concerning the mechanical aspects of the gait process. The study of mechanical energy in the lower limbs allowed for a better understanding of the recovery of the lower limbs during walking in patients after TKA or UKA. This would help clinicians and therapists to design rehabilitation programme so that patients after TKA or UKA can improve to normal levels more quickly and fully.

In this study, the research hypotheses were that there was a difference in lower limb energy use between operated and non-operated side 6 months after TKA or UKA, and the lower limb energy performance in operated side was decreased. The purpose of this study was to prospectively evaluate the walking energy performance of patients who had undergone TKA or UKA compared to control group without surgery. The study would also compare the energy performances between the operated and non-operated sides. Clinically, this study would contribute new knowledge on how patients use energies during walking, which would help clinicians and patients to arrange relevant exercises during rehabilitation. In methodology, this project made a useful computer program which could be used in clinical gait analysis for limb energy.

## 2 Methods

### 2.1 Patient involvement

A total of 8 post-UKA patients, 10 post-TKA patients, and 11 individuals who had not undergone knee joint surgery were collected from the existing database in our lab. Due to Covid situations, the number of participants could not be increased. When data collection, the inclusion was: 1) those undergone a UKA or TKA for knee osteoarthritis in the local region; 2) those had not undergone knee joint surgery as control; 3) those have an asymptomatic/clinically normal contralateral knee and be able to walk without a walking aid. Exclusion was: 1) those suffered from any poly-arthropathy; 2) suffered from any neuromuscular disease or cardiovascular disorders. All participants signed a written consent form when data collection. The ethical approval was shown in the cover letter for reviewing stage. As the gait data for this study was collected from the existing database, a Caldicott approval was waived from the School of Medicine Research Ethics Committee.

### 2.2 Laboratory and Instrumentation

The original gait data for the knee replacement was collected at our lab. The lab is 18 m long and 9 m wide enough for any motion measurement, equipped with eighteen highly specialized Vicon optoelectronic cameras (Oxford, UK).

### 2.3 Data collection

Twenty reflex markers were placed on both lower limbs and the pelvis for each participant as shown in Table 1. A static trial called T-pose was collected so that Vicon Plug-in-Gait model calculated skeletal dimensions. The participants were asked to walk in their natural way as everyday life. Most participants were asked to repeat the walking multiple times to make sure the entire cycle with good marker quality. Finally, 3 good trials for each participant were used in the analysis. All trails were labelled using Vicon Nexus Plug-in-Gait model to obtain the 3D coordinates for the markers and joint centres. The markers and joint centres were used to calculate CoM for each segment, and further used to estimate the moment of inertia for each segment. The gait cycles were manually defined as three events, foot strike, foot off and foot strike again. Only the defined cycles were analysed.

**Table 1:** Anatomical sites of markers.

Name of markers	Anatomical sites
LASI	Placed directly over the left anterior superior iliac spine
RASI	Placed directly over the right anterior superior iliac spine
LPSI	Placed directly over the left posterior superior iliac spine
RPSI	Placed directly over the right posterior superior iliac spine
SACR	Placed on the skin mid-way between the posterior superior iliac spines (PSIS). An alternative to LPSI and RPSI
THI	Place the marker over the lower lateral 1/3 surface of the thigh, just below the swing of the hand
LANK	Placed on the lateral malleolus along an imaginary line that passes through the transmalleolar axis
LTIB	Like the thigh markers, these are placed over the lower 1/3 of the shank to determine the alignment of the ankle flexion axis
LTOE	Placed over the second metatarsal head, on the mid-foot side of the equinus break between forefoot and mid-foot
LHEE	Placed on the calcaneus at the same height above the plantar surface of the foot as the toe marker

### 2.4 Energy calculation

In motion analysis, mechanical energy is often divided into potential energy (PE) and kinetic energy (KE). The formulas for energy calculation are shown in the following equations (1), (2), (3):

(1) PE=mgh

Firstly, PE is potential energy, the product of the mass and the height of the centre of mass (CoM), where m is the mass of an object, g is the gravitational constant, approximately 9.81 m/s^2^, and h is the height of the CoM in a segment relative to a ground level. From the marker coordinates, the mass and CoM were calculated for each segment or the entire lower limb, based on the relative length of the segments and the relative mass of the whole body [[15], [16], [17], [18]].

(2) $$\text{KE}=\frac{1}{2}mv^{2}$$

KE is kinetic energy, the product of the mass and the square of the velocity of CoM.

(3) $$\text{RKE=}\frac{1}{2}{\text{I}}_{c}\omega ²$$

Where RKE is the rotational kinetic energy, *I*_*c*_ is the moment of inertia of the segment about its CoM, with units of kgm^2^, and ω the angular velocity of the segment (rad/s).

(4) $$\text{Recovery}\;\text{Coef}=\frac{\left(\Delta \text{PE}+\Delta \text{KE}\right)-\Delta \left(\text{PE}+\text{KE}\right)}{\left(\Delta \text{PE}+\Delta \text{KE}\right)}$$

Recovery Coefficient is the ration of the changes of kinetic energy and potential energy to the change of total energy as shown in equation (4) [19], where ΔPE is the maximum change in the potential energy; ΔKE the maximum change in the kinetic energy; and Δ(PE + KE) the maximum change in the sum of the two energies. This coefficient shows the amount of how much the potential and kinetic energies are exchanged between each other. The coefficient value is within 0–100%, and the higher, the better.

The calculation of energy was for the upper leg, lower leg, and foot. Whole lower limb including three segments was also considered as a single multi-segment system for energy analysis. Further, the centre of pelvis constructed by using the centre of four markers, LASI, RASI, LPSI and RPSI (Table 1) was considered as the approximate to the CoM of whole body, and thus the centre of pelvis was calculated for energy change as whole body.

### 2.5 Statistical analysis

Mean and standard deviation were calculated for all demographic characteristics (Mass, BMI, Height, Segment Length). This study utilized SPSS® version 28 (SPSS® Inc., Chicago, IL, USA) for statistical data analysis. The TKA and UKA groups were divided into operated side and non-operated side based on whether the knee joint was operated. The control group was categorized with the left leg as the assumed operated side and the right leg as the non-operated side. Subsequently, splitting the data according to the operated and non-operated sides was a necessary step to obtain clear results for each side during the gait analysis. The data was analysed using the Repeated Measures or Multivariate options, which is derived from the General Linear Model in SPSS. This approach allowed users not only to input of repeated measurements for paired variable and independent variables for comparison, but also input multi fixed or interactive factors in statistical analysis. In statistical analysis, the operative-sides and non-operative-sides were compared as within-subject way, and the patient and control groups were compared as between-subject way. Additionally, other factors such as Sex was included as fixed factors, while BMI and Age were incorporated as covariate factors. The parameters being compared were set as dependent variables. This facilitated the examination of differences between the operated and non-operated sides in the three groups, displaying means according to the selected factors. A significance level of p < 0.05 was adopted as the threshold for statistical significance.

As this study is completely new and there were no reference studies, there was no power analysis done. The sample size used included all samples available in the database.

## 3 Results

### 3.1 Demography parameters

The demographic detail is shown in Table 2. In the TKA group, there were a total of 10 volunteers, including 4 females and 6 males. The participants had an average height of 1.68 (±0.10) m and a weight of 70.91(±8.0) kg. There were 8 volunteers, with 3 females and 5 males in UKA. The average height of the participants was 1.65(±0.09) m, and their weight was 74.14 (±8.92) kg. The Control group consisted of 11 volunteers, including 3 females and 8 males. The participants had an average height of 1.71 (±0.97) m and a weight of 75.66 (±8.07) kg. Demographic information and clinical condition for the participants were shown in Table 2. Overall, the heights are not significantly different among groups.

**Table 2:** Demography information for the participants.

Dependent Variable	CG (n = 11)	TKA (n = 10)	UKA (n = 8)	Sig.
Continuous variables	Mean ± SD			ANOVA
BMI	25.79 ± 1.35	32.30 ± 3.84	27.21 ± 3.17	*p* < 0.001
Body mass (Kg)	75.66 ± 8.07	70.91 ± 8.00	74.14 ± 8.92	*p* < 0.001
Height (m)	1.71 ± 0.97	1.68 ± 0.10	1.65 ± 0.09	0.06
Age (years)	55.00 ± 2.87	68.80 ± 6.05	69.89 ± 7.35	*p* < 0.001
Categorical variable	%			Chi-Square
Male	72.72%	60.00%	62.50%	0.811

Note: CG: Control group.

### 3.2 Gait parameters

The gait parameters are reported in Table 3. Based on the data from the three groups of participants, it was found that the cadence in the Control group was significantly higher than that in the TKA and UKA groups. Additionally, in the comparison of the operated sides among the three groups, the Control group exhibited a significantly faster walking speed compared to the TKA. No significant difference was observed in other comparisons.

**Table 3:** Comparison of gait parameters among the TKA, UKA and Control groups.

Dependent Variable	Group	Mean ± SE	95% Confidence Interval		Notes
			Lower Bound	Upper Bound	
Cadence 1 （step/min)	CG	118.44 ± 3.34	111.76	125.11	CG vs TKA*
	TKA	99.67 ± 3.72	92.23	107.11	CG vs UKA**
	UKA	100.51 ± 3.28	93.95	107.06	TKA vs UKA
Cadence 2 （step/min)	CG	117.61 ± 3.09	111.45	123.77	CG vs TKA**
	TKA	99.80 ± 3.44	92.93	106.68	CG vs UKA**
	UKA	101.09 ± 3.03	95.04	107.15	TKA vs UKA
Walking speed 1 (m/s)	CG	1.20 ± 0.05	1.10	1.30	CG vs TKA
	TKA	1.05 ± 0.06	0.94	1.16	CG vs UKA
	UKA	1.10 ± 0.05	1.00	1.20	TKA vs UKA
Walking speed 2 (m/s)	CG	1.26 ± 0.06	1.14	1.38	CG vs TKA*
	TKA	0.94 ± 0.07	0.81	1.08	CG vs UKA
	UKA	1.09 ± 0.06	0.97	1.20	TKA vs UKA
Stride length 1 (m)	CG	1.21 ± 0.03	1.15	1.28	CG vs TKA
	TKA	1.26 ± 0.04	1.19	1.33	CG vs UKA
	UKA	1.30 ± 0.03	1.23	1.36	TKA vs UKA
Stride length 2 (m)	CG	1.32 ± 0.06	1.21	1.43	CG vs TKA
	TKA	1.10 ± 0.06	0.98	1.22	CG vs UKA
	UKA	1.27 ± 0.05	1.16	1.38	TKA vs UKA
Step length 1 (m)	CG	0.61 ± 0.02	0.58	0.65	CG vs TKA
	TKA	0.61 ± 0.02	0.57	0.65	CG vs UKA
	UKA	0.65 ± 0.02	0.62	0.69	TKA vs UKA
Step length 2 (m)	CG	0.66 ± 0.03	0.60	0.71	CG vs TKA
	TKA	0.56 ± 0.03	0.50	0.63	CG vs UKA
	UKA	0.64 ± 0.03	0.58	0.70	TKA vs UKA

Notes: a Covariates appearing in the model are evaluated at the following values: Age = 62.66， BMI = 27.98.

b Adjustment for multiple comparisons: Bonferroni.

c Dependent Variable 1: Leg without surgical operation; Dependent Variable 2: Leg with surgical operation. KE is translational kinetic energy; PE is potential energy; CG is control group. *：*p*＜0.05; **: *p* ＜0.01; ***: *p* ＜0.001.

### 3.3 Kinetic energy parameters

#### 3.3.1 Control group versus TKA group

The following results were observed when comparing the different segments of the lower limbs individually: in the comparison of the operated side of the lower leg, the Control group's Min KE is significantly lower than that in the TKA. In the comparison of the operated side of the foot segment, both the Max KE and the range of KE of the Control group are significantly lower than those of the TKA as in Table 4 and Table 5.

**Table 4:** Comparison of Kinetic and Potential energy (Joule) parameters and recovery coefficients of the whole lower limb among the TKA, UKA, and Control groups.

Segment	Dependent Variable	Group	Mean ± SE	95% Confidence Interval		Notes
				Lower Bound	Upper Bound	
The whole lower limb	MaxKE.1 (Joule)	CG	26.74 ± 2.17	22.41	31.07	CG vs TKA
		TKA	22.31 ± 2.42	17.48	27.14	CG vs UKA
		UKA	25.24 ± 2.13	20.98	29.49	TKA vs UKA
	maxKE.2 (Joule)	CG	25.66 ± 2.13	21.42	29.91	CG vs TKA
		TKA	26.61 ± 2.37	21.87	31.34	CG vs UKA
		UKA	27.1 ± 2.09	22.92	31.27	TKA vs UKA
	MinKE.1 (Joule)	CG	1.52 ± 0.14	1.25	1.79	CG vs TKA
		TKA	1.43 ± 0.15	1.13	1.73	CG vs UKA
		UKA	1.52 ± 0.13	1.26	1.79	TKA vs UKA
	MinKE.2 (Joule)	CG	1.43 ± 0.15	1.13	1.73	CG vs TKA
		TKA	1.57 ± 0.17	1.24	1.91	CG vs UKA
		UKA	1.69 ± 0.15	1.39	1.98	TKA vs UKA
	RangeKE.1 (Joule)	CG	25.23 ± 2.06	21.11	29.34	CG vs TKA
		TKA	20.88 ± 2.30	16.29	25.47	CG vs UKA
		UKA	23.71 ± 2.02	19.67	27.76	TKA vs UKA
	RangeKE.2 (Joule)	CG	24.23 ± 2.02	20.21	28.26	CG vs TKA
		TKA	25.04 ± 2.25	20.55	29.53	CG vs UKA
		UKA	25.41 ± 1.98	21.45	29.37	TKA vs UKA
	MaxPE.1 (Joule)	CG	63.27 ± 2.24	58.79	67.74	CG vs TKA***
		TKA	80.56 ± 2.50	75.56	85.55	CG vs UKA***
		UKA	78.32 ± 2.20	73.92	82.72	TKA vs UKA
	MaxPE.2 (Joule)	CG	63.48 ± 2.23	59.01	67.94	CG vs TKA***
		TKA	80.49 ± 2.49	75.52	85.47	CG vs UKA***
		UKA	77.09 ± 2.20	72.70	81.47	TKA vs UKA
	MinPE.1 (Joule)	CG	58.50 ± 2.03	54.44	62.55	CG vs TKA***
		TKA	74.47 ± 2.26	69.95	78.99	CG vs UKA***
		UKA	72.28 ± 1.99	68.30	76.27	TKA vs UKA
	MinPE.2 (Joule)	CG	58.93 ± 2.01	54.90	62.96	CG vs TKA***
		TKA	73.90 ± 2.25	69.42	78.39	CG vs UKA**
		UKA	71.28 ± 1.98	67.33	75.24	TKA vs UKA
	RangePE.1 (Joule)	CG	4.77 ± 0.32	4.12	5.42	CG vs TKA
		TKA	6.08 ± 0.36	5.36	6.80	CG vs UKA
		UKA	6.04 ± 0.32	5.40	6.68	TKA vs UKA
	RangePE.2 (Joule)	CG	4.55 ± 0.35	3.85	5.24	CG vs TKA
		TKA	6.59 ± 0.39	5.81	7.37	CG vs UKA
		UKA	5.80 ± 0.34	5.12	6.49	TKA vs UKA
	RecoveryCof.1	CG	8.62 ± 1.01	6.61	10.63	CG vs TKA
		TKA	10.73 ± 1.12	8.49	12.97	CG vs UKA
		UKA	9.02 ± 0.99	7.04	10.99	TKA vs UKA
	RecoveryCof.2	CG	9.68 ± 0.79	8.09	11.27	CG vs TKA
		TKA	8.88 ± 0.89	7.11	10.65	CG vs UKA
		UKA	7.54 ± 0.78	5.97	9.10	TKA vs UKA

Note: a. Covariates appearing in the model are evaluated at the following values: Age = 62.66， BMI = 27.98.

b. Adjustment for multiple comparisons: Bonferroni.

c.Dependent Variable 1: Leg without surgical operation; Dependent Variable 2: Leg with surgical operation. KE is translational kinetic energy; PE is potential energy; CG is control group. *：*p*＜0.05; **: *p* ＜0.01; ***: *p* ＜0.001.

**Table 5:** Comparison of Kinetic and Potential energy (Joule) parameters of the segments among the TKA, UKA, and Control groups.

Segment	Dependent Variable	Group	Mean ± SE	95% Confidence Interval		Notes
				Lower Bound	Upper Bound	
**Upper leg**	**MaxKE.1 (Joule)**	**CG**	**12.42 ± 0.99**	**10.45**	**14.39**	**CG vs TKA**
		**TKA**	**11.00 ± 1.10**	**8.80**	**13.19**	**CG vs UKA**
		**UKA**	**12.67 ± 0.97**	**10.74**	**14.61**	**TKA vs UKA**
	**MaxKE.2 (Joule)**	**CG**	**12.99 ± 0.91**	**11.18**	**14.81**	**CG vs TKA**
		**TKA**	**10.53 ± 1.01**	**8.50**	**12.55**	**CG vs UKA**
		**UKA**	**12.26 ± 0.89**	**10.47**	**14.04**	**TKA vs UKA**
	**MinKE.1 (Joule)**	**CG**	**2.02 ± 0.18**	**1.66**	**2.38**	**CG vs TKA**
		**TKA**	**1.86 ± 0.20**	**1.47**	**2.26**	**CG vs UKA**
		**UKA**	**1.99 ± 0.18**	**1.64**	**2.34**	**TKA vs UKA**
	**MinKE.2 (Joule)**	**CG**	**1.95 ± 0.19**	**1.57**	**2.33**	**CG vs TKA**
		**TKA**	**1.97 ± 0.21**	**1.54**	**2.39**	**CG vs UKA**
		**UKA**	**2.15 ± 0.19**	**1.78**	**2.53**	**TKA vs UKA**
	**RangeKE.1 (Joule)**	**CG**	**10.40 ± 0.84**	**8.72**	**12.07**	**CG vs TKA**
		**TKA**	**9.13 ± 0.94**	**7.26**	**11.00**	**CG vs UKA**
		**UKA**	**10.68 ± 0.82**	**9.03**	**12.33**	**TKA vs UKA**
	**RangeKE.2 (Joule)**	**CG**	**11.05 ± 0.75**	**9.56**	**12.54**	**CG vs TKA**
		**TKA**	**8.56 ± 0.83**	**6.90**	**10.22**	**CG vs UKA**
		**UKA**	**10.10 ± 0.73**	**8.64**	**11.57**	**TKA vs UKA**
	**MaxPE.1 (Joule)**	**CG**	**51.27 ± 1.77**	**47.73**	**54.81**	CG vs TKA***
		**TKA**	**65.82 ± 1.98**	**61.87**	**69.77**	CG vs UKA***
		**UKA**	**63.66 ± 1.74**	**60.17**	**67.14**	**TKA vs UKA**
	**MaxPE.2 (Joule)**	**CG**	**51.59 ± 1.79**	**48.02**	**55.16**	CG vs TKA***
		**TKA**	**65.41 ± 1.99**	**61.43**	**69.39**	CG vs UKA***
		**UKA**	**62.89 ± 1.76**	**59.38**	**66.40**	**TKA vs UKA**
	**MinPE.1 (Joule)**	**CG**	**48.13 ± 1.65**	**44.83**	**51.43**	CG vs TKA***
		**TKA**	**61.88 ± 1.84**	**58.20**	**65.56**	CG vs UKA***
		**UKA**	**59.68 ± 1.62**	**56.44**	**62.93**	**TKA vs UKA**
	**MinPE.2 (Joule)**	**CG**	**48.41 ± 1.67**	**45.07**	**51.75**	CG vs TKA***
		**TKA**	**61.35 ± 1.86**	**57.63**	**65.07**	CG vs UKA***
		**UKA**	**58.84 ± 1.64**	**55.56**	**62.13**	**TKA vs UKA**
	**RangePE.1 (Joule)**	**CG**	**3.14 ± 0.26**	**2.61**	**3.66**	**CG vs TKA**
		**TKA**	**3.93 ± 0.29**	**3.35**	**4.52**	**CG vs UKA**
		**UKA**	**3.97 ± 0.26**	**3.46**	**4.49**	**TKA vs UKA**
	**RangePE.2 (Joule)**	**CG**	**3.18 ± 0.26**	**2.67**	**3.70**	**CG vs TKA**
		**TKA**	**4.07 ± 0.29**	**3.49**	**4.64**	**CG vs UKA**
		**UKA**	**4.05 ± 0.25**	**3.54**	**4.55**	**TKA vs UKA**
**Lower leg**	**MaxKE.1 (Joule)**	**CG**	**10.96 ± 0.86**	**9.24**	**12.68**	**CG vs TKA**
		**TKA**	**9.02 ± 0.96**	**7.09**	**10.94**	**CG vs UKA**
		**UKA**	**10.05 ± 0.85**	**8.36**	**11.75**	**TKA vs UKA**
	**MaxKE.2 (Joule)**	**CG**	**10.99 ± 0.85**	**9.28**	**12.69**	**CG vs TKA**
		**TKA**	**10.07 ± 0.95**	**8.17**	**11.97**	**CG vs UKA**
		**UKA**	**10.84 ± 0.84**	**9.16**	**12.51**	**TKA vs UKA**
	**MinKE.1 (Joule)**	**CG**	**0.04 ± 0.01**	**0.03**	**0.05**	**CG vs TKA**
		**TKA**	**0.05 ± 0.01**	**0.03**	**0.06**	**CG vs UKA**
		**UKA**	**0.05 ± 0.01**	**0.04**	**0.06**	**TKA vs UKA**
	**MinKE.2 (Joule)**	**CG**	**0.03 ± 0.01**	**0.01**	**0.04**	CG vs TKA*
		**TKA**	**0.07 ± 0.01**	**0.05**	**0.09**	CG vs UKA**
		**UKA**	**0.07 ± 0.01**	**0.05**	**0.09**	**TKA vs UKA**
	**RangeKE.1 (Joule)**	**CG**	**10.92 ± 0.86**	**9.20**	**12.64**	**CG vs TKA**
		**TKA**	**8.97 ± 0.96**	**7.05**	**10.89**	**CG vs UKA**
		**UKA**	**10.00 ± 0.85**	**8.31**	**11.69**	**TKA vs UKA**
	**RangeKE.2 (Joule)**	**CG**	**10.96 ± 0.85**	**9.26**	**12.65**	**CG vs TKA**
		**TKA**	**10.00 ± 0.95**	**8.11**	**11.89**	**CG vs UKA**
		**UKA**	**10.77 ± 0.83**	**9.10**	**12.43**	**TKA vs UKA**
	**MaxPE.1 (Joule)**	**CG**	**10.65 ± 0.41**	**9.83**	**11.47**	CG vs TKA**
		**TKA**	**13.13 ± 0.46**	**12.22**	**14.05**	CG vs UKA**
		**UKA**	**12.89 ± 0.40**	**12.08**	**13.70**	**TKA vs UKA**
	**MaxPE.2 (Joule)**	**CG**	**10.76 ± 0.40**	**9.97**	**11.55**	CG vs TKA**
		**TKA**	**13.07 ± 0.44**	**12.19**	**13.95**	CG vs UKA*
		**UKA**	**12.65 ± 0.39**	**11.87**	**13.42**	**TKA vs UKA**
	**MinPE.1 (Joule)**	**CG**	**8.89 ± 0.33**	**8.24**	**9.54**	CG vs TKA**
		**TKA**	**10.88 ± 0.36**	**10.16**	**11.61**	CG vs UKA**
		**UKA**	**10.68 ± 0.32**	**10.04**	**11.32**	**TKA vs UKA**
	**MinPE.2 (Joule)**	**CG**	**8.96 ± 0.32**	**8.33**	**9.59**	CG vs TKA**
		**TKA**	**10.79 ± 0.35**	**10.09**	**11.50**	CG vs UKA**
		**UKA**	**10.56 ± 0.31**	**9.94**	**11.18**	**TKA vs UKA**
	**RangePE.1 (Joule)**	**CG**	**1.76 ± 0.11**	**1.55**	**1.97**	CG vs TKA*
		**TKA**	**2.25 ± 0.12**	**2.02**	**2.48**	CG vs UKA*
		**UKA**	**2.21 ± 0.10**	**2.00**	**2.41**	**TKA vs UKA**
	**RangePE.2 (Joule)**	**CG**	**1.80 ± 0.10**	**1.59**	**2.01**	CG vs TKA*
		**TKA**	**2.28 ± 0.12**	**2.05**	**2.51**	**CG vs UKA**
		**UKA**	**2.09 ± 0.10**	**1.88**	**2.29**	**TKA vs UKA**
**Foot**	**MaxKE.1 (Joule)**	**CG**	**11.63 ± 0.87**	**9.89**	**13.36**	**CG vs TKA**
		**TKA**	**8.97 ± 0.97**	**7.03**	**10.90**	**CG vs UKA**
		**UKA**	**9.87 ± 0.85**	**8.17**	**11.57**	**TKA vs UKA**
	**MaxKE.2 (Joule)**	**CG**	**21.71 ± 14.70**	**7.65**	**51.08**	CG vs TKA**
		**TKA**	**66.35 ± 16.38**	**33.61**	**99.08**	**CG vs UKA**
		**UKA**	**24.04 ± 14.44**	**4.82**	**52.90**	**TKA vs UKA**
	**MinKE.1 (Joule)**	**CG**	**0.00 ± 0.00**	**0.00**	**0.00**	**CG vs TKA**
		**TKA**	**0.00 ± 0.00**	**0.00**	**0.00**	**CG vs UKA**
		**UKA**	**0.00 ± 0.00**	**0.00**	**0.00**	**TKA vs UKA**
	**MinKE.2 (Joule)**	**CG**	**0.00 ± 0.00**	**0.00**	**0.00**	**CG vs TKA**
		**TKA**	**0.00 ± 0.00**	**0.00**	**0.00**	**CG vs UKA**
		**UKA**	**0.00 ± 0.00**	**0.00**	**0.00**	**TKA vs UKA**
	**RangeKE.1 (Joule)**	**CG**	**11.63 ± 0.87**	**9.89**	**13.36**	**CG vs TKA**
		**TKA**	**8.97 ± 0.97**	**7.03**	**10.90**	**CG vs UKA**
		**UKA**	**9.87 ± 0.85**	**8.17**	**11.57**	**TKA vs UKA**
	**RangeKE.2 (Joule)**	**CG**	**21.71 ± 14.70**	**−51.08**	**7.65**	CG vs TKA**
		**TKA**	**66.35 ± 16.38**	**33.61**	**99.08**	**CG vs UKA**
		**UKA**	**24.04 ± 14.44**	**−4.82**	**52.90**	**TKA vs UKA**
	**MaxPE.1 (Joule)**	**CG**	**2.30 ± 0.13**	**2.05**	**2.56**	CG vs TKA*
		**TKA**	**2.88 ± 0.14**	**2.60**	**3.17**	CG vs UKA**
		**UKA**	**3.05 ± 0.12**	**2.81**	**3.30**	**TKA vs UKA**
	**MaxPE.2 (Joule)**	**CG**	**2.26 ± 0.09**	**2.08**	**2.43**	CG vs TKA***
		**TKA**	**3.05 ± 0.10**	**2.86**	**3.25**	CG vs UKA***
		**UKA**	**2.95 ± 0.09**	**2.77**	**3.12**	**TKA vs UKA**
	**MinPE.1 (Joule)**	**CG**	**0.82 ± 0.05**	**0.72**	**0.92**	CG vs TKA***
		**TKA**	**1.17 ± 0.05**	**1.06**	**1.27**	CG vs UKA***
		**UKA**	**1.22 ± 0.05**	**1.13**	**1.32**	**TKA vs UKA**
	**MinPE.2 (Joule)**	**CG**	**0.83 ± 0.04**	**0.74**	**0.91**	CG vs TKA***
		**TKA**	**1.20 ± 0.05**	**1.10**	**1.30**	CG vs UKA***
		**UKA**	**1.16 ± 0.04**	**1.07**	**1.24**	**TKA vs UKA**
	**RangePE.1 (Joule)**	**CG**	**1.48 ± 0.09**	**1.31**	**1.66**	**CG vs TKA**
		**TKA**	**1.72 ± 0.10**	**1.52**	**1.91**	**CG vs UKA**
		**UKA**	**1.83 ± 0.09**	**1.66**	**2.00**	**TKA vs UKA**
	**RangePE.2 (Joule)**	**CG**	**1.43 ± 0.06**	**1.32**	**1.54**	CG vs TKA***
		**TKA**	**1.85 ± 0.06**	**1.73**	**1.98**	CG vs UKA***
		**UKA**	**1.79 ± 0.06**	**1.68**	**1.90**	**TKA vs UKA**

Note: a Covariates appearing in the model are evaluated at the following values: Age = 62.66， BMI = 27.98.

b Adjustment for multiple comparisons: Bonferroni. c Dependent Variable 1: Leg without surgical operation; Dependent Variable 2: Leg with surgical operation. KE is translational kinetic energy; PE is potential energy; CG is control group. *：*p*＜0.05; **: *p* ＜0.01; ***: *p* ＜0.001.

**Fig. 1:**
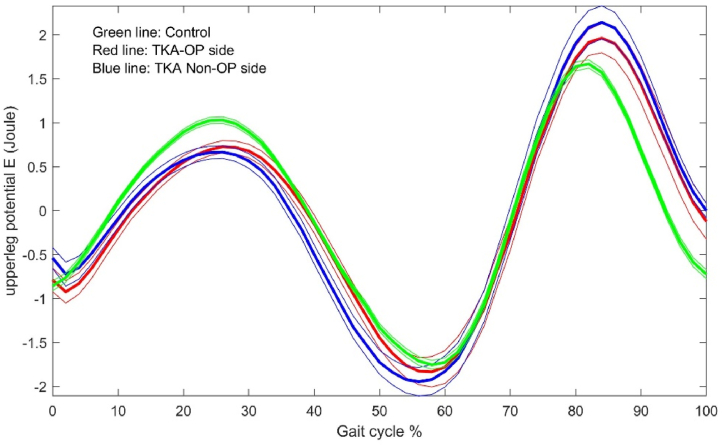
Comparison of potential energies between TKE op- and non-op sides and control groups in the upper leg. Green-control, red-TKE op-side, blue- TKE non-op side. (For interpretation of the references to colour in this figure legend, the reader is referred to the Web version of this article.)

**Fig. 2:**
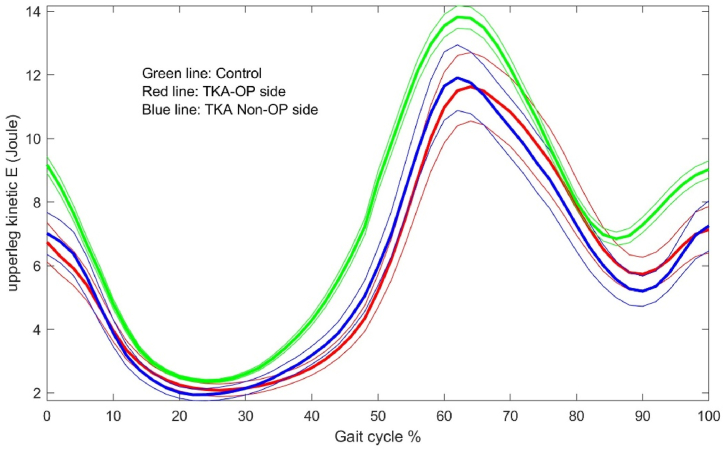
Comparison of kinetic energies between TKE op- and non-op sides and control groups in the upper leg. Green-control, red-TKE op-side, blue- TKE non-op side. (For interpretation of the references to colour in this figure legend, the reader is referred to the Web version of this article.)

**Fig. 3:**
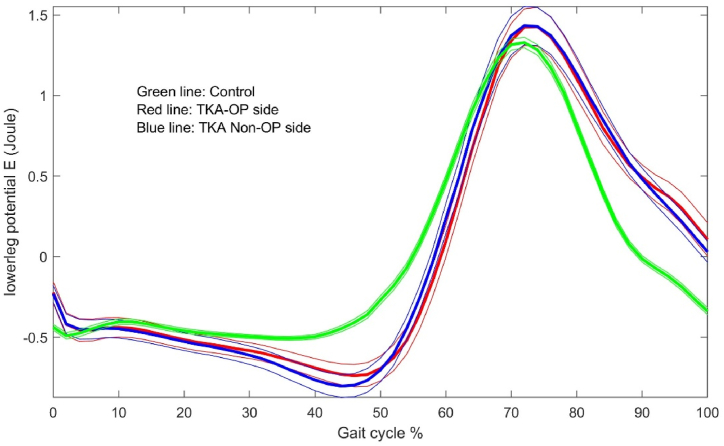
Comparison of potential energies between TKE op- and non-op sides and control groups in the lower leg.

**Fig. 4:**
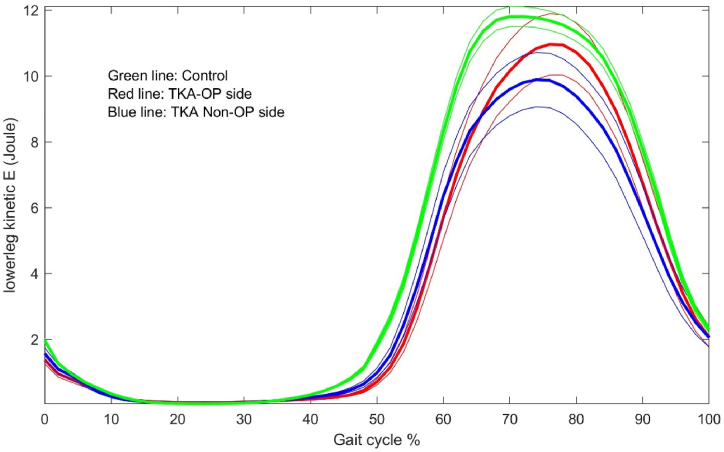
Comparison of kinetic energies between TKE op- and non-op sides and control groups in the lower leg.

**Fig. 5:**
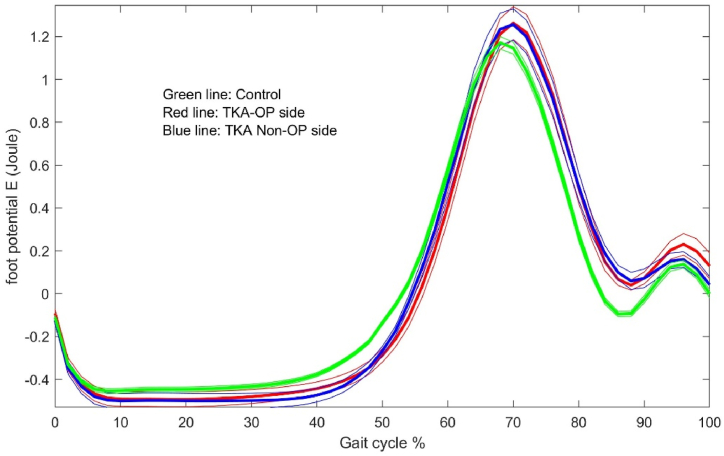
Comparison of potential energies between TKE op- and non-op sides and control groups in the foot.

**Fig. 6:**
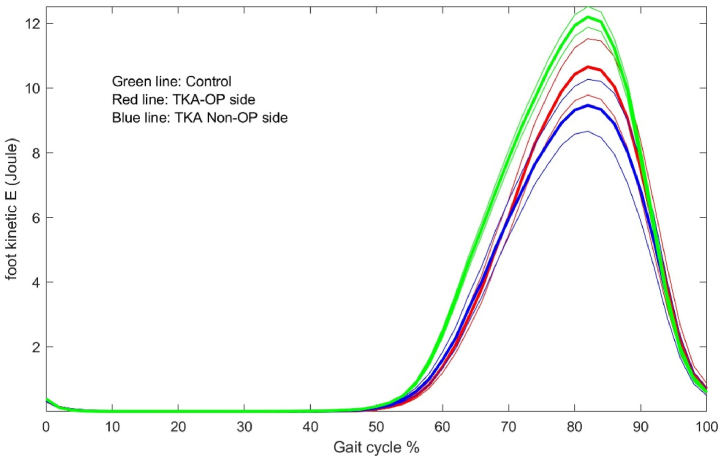
Comparison of kinetic energies between TKE op- and non-op sides and control groups in the foot.

The energy dynamic changes in the upper leg, lower leg and foot are shown in Fig. 1, Fig. 2, Fig. 3, Fig. 4, Fig. 5, Fig. 6. As the UKA group has similar trends to the TKA, the figures for the UKA are neglected.

#### 3.3.2 Control group versus UKA group

The results showed that the operated side of UKA exhibited significantly higher Min KE than the Control group in the lower leg segment. However, there was no significant difference observed in other aspects of comparison between the two groups in Table 4 and Table 5.

#### 3.3.3 TKA group versus UKA group

The results are shown in Table 4, Table 5 No significant difference was found in KE between TKA and UKA.

#### 3.3.4 Non-operated side versus operated side

When compared whole lower limb, the Min KE in operated side was significantly higher than non-operated side in UKA. The Max KE and the range of KE in non-operated side was lower than operated side in TKA (Table 6). In the lower leg of the TKA group, the range of KE and Max KE were significantly higher on the operated side than on the non-operated side. The operated side had significantly higher Min KE than the non-operated side in the foot of TKA. In the upper leg and lower leg of the UKA group, the Min KE is significantly higher on the operated side than on the non-operated side. In physics, the change in energy is roughly equal to the work output by an object, so the non-operative side of the lower leg in the TKA group did less work in gait than the operative side as shown in Table 7.

**Table 6:** Comparison of Kinetic and Potential energy (Joule) parameters and Recovery Coefficients of the whole between operated side and non-operated side.

Segment	Group	Dependent Variable	Mean ± SE	95% Confidence Interval	
				Lower Bound	Upper Bound
**The whole lower limb**	**CG**	**MaxKE.1(Joule)**	**29.76 ± 1.09**	**27.55**	**31.98**
		**MaxKE.2(Joule)**	**30.13 ± 1.13**	**27.82**	**32.44**
		**MinKE.1(Joule)**	**1.77 ± 0.06**	**1.64**	**1.90**
		**MinKE.2(Joule)**	**1.69 ± 0.05**	**1.59**	**1.79**
		**RangeKE.1(Joule)**	**28.00 ± 1.04**	**25.88**	**30.12**
		**RangeKE.2(Joule)**	**28.44 ± 1.10**	**26.21**	**30.68**
		**MaxPE.1(Joule)**	**70.69 ± 1.80**	**67.02**	**74.36**
		**MaxPE.2(Joule)**	**70.58 ± 1.84**	**66.84**	**74.32**
		**MinPE.1(Joule)**	**65.42 ± 1.70**	**61.95**	**68.88**
		**MinPE.2(Joule)**	**65.43 ± 1.71**	**61.94**	**68.92**
		**RangePE.1(Joule)**	**5.27 ± 0.14**	**4.98**	**5.56**
		**RangePE.2(Joule)**	**5.15 ± 0.18**	**4.78**	**5.52**
		**RecoveryCof.1**	8.69 ± 0.34*	**8.00**	**9.39**
		**RecoveryCof.2**	9.53 ± 0.42*	**8.67**	**10.39**
	**TKA**	**MaxKE.1(Joule)**	24.98 ± 2.48*	**19.80**	**30.17**
		**MaxKE.2(Joule)**	28.49 ± 2.78*	**22.68**	**34.30**
		**MinKE.1(Joule)**	**1.51 ± 0.16**	**1.17**	**1.84**
		**MinKE.2(Joule)**	**1.59 ± 0.18**	**1.21**	**1.97**
		**RangeKE.1(Joule)**	23.47 ± 2.33*	**18.59**	**28.36**
		**RangeKE.2(Joule)**	26.90 ± 2.63*	**21.40**	**32.40**
		**MaxPE.1(Joule)**	**85.74 ± 4.25**	**76.84**	**94.63**
		**MaxPE.2(Joule)**	**86.39 ± 4.39**	**77.20**	**95.58**
		**MinPE.1(Joule)**	**78.95 ± 3.77**	**71.07**	**86.83**
		**MinPE.2(Joule)**	**79.30 ± 3.94**	**71.05**	**87.54**
		**RangePE.1(Joule)**	**6.78 ± 0.52**	**5.69**	**7.88**
		**RangePE.2(Joule)**	**7.10 ± 0.51**	**6.02**	**8.17**
		**RecoveryCof.1**	**9.94 ± 1.46**	**6.89**	**12.99**
		**RecoveryCof.2**	**8.50 ± 0.67**	**7.10**	**9.90**
	**UKA**	**MaxKE.1(Joule)**	**22.04 ± 2.50**	**16.76**	**27.32**
		**MaxKE.2(Joule)**	**22.25 ± 2.25**	**17.49**	**27.00**
		**MinKE.1(Joule)**	1.22 ± 0.14***	**0.92**	**1.51**
		**MinKE.2(Joule)**	1.40 ± 0.15***	**1.09**	**1.72**
		**RangeKE.1(Joule)**	**20.82 ± 2.38**	**15.80**	**25.85**
		**RangeKE.2(Joule)**	**20.84 ± 2.13**	**16.35**	**25.33**
		**MaxPE.1(Joule)**	70.38 ± 2.64**	**64.80**	**75.95**
		**MaxPE.2(Joule)**	69.34 ± 2.66**	**63.72**	**74.95**
		**MinPE.1(Joule)**	65.14 ± 2.34*	**60.21**	**70.07**
		**MinPE.2(Joule)**	64.22 ± 2.39*	**59.18**	**69.25**
		**RangePE.1(Joule)**	**5.24 ± 0.39**	**4.41**	**6.06**
		**RangePE.2(Joule)**	**5.12 ± 0.36**	**4.37**	**5.87**
		**RecoveryCof.1**	8.74 ± 0.51**	**7.66**	**9.82**
		**RecoveryCof.2**	7.67 ± 0.60**	**6.41**	**8.94**

Note: Dependent Variable 1: Leg without surgical operation; Dependent Variable 2: Leg with surgical operation. KE is translational kinetic energy; PE is potential energy; CG is control group. *：*p*＜0.05; **: *p* ＜0.01; ***: *p* ＜0.001.

**Table 7:** Comparison of kinetic and potential energy (Joule) parameters of the segments between operated side and non-operated side.

Segment	Group	Dependent Variable	Type	Mean ± SD	95% Confidence Interval	
					Lower Bound	Upper Bound
**Upper leg**	**CG**	**MaxKE(Joule)**	**Opt-no**	13.83 ± 0.47*	**12.84**	**14.82**
			**Opt-yes**	14.23 ± 0.52*		
		**MinKE(Joule)**	**Opt-no**	**2.36 ± 0.09**	**2.21**	**2.52**
			**Opt-yes**	**2.28 ± 0.07**		
		**RangeKE(Joule)**	**Opt-no**	11.46 ± 0.40*	**10.60**	**12.33**
			**Opt-yes**	11.94 ± 0.46*		
		**MaxPE(Joule)**	**Opt-no**	**57.44 ± 1.46**	**54.50**	**60.37**
			**Opt-yes**	**57.38 ± 1.48**		
		**MinPE(Joule)**	**Opt-no**	53.96 ± 1.40*	**51.16**	**56.75**
			**Opt-yes**	53.72 ± 1.40*		
		**RangePE(Joule)**	**Opt-no**	3.48 ± 0.12*	**3.24**	**3.72**
			**Opt-yes**	3.66 ± 0.12*		
	**TKA**	**MaxKE(Joule)**	**Opt-no**	**12.38 ± 1.26**	**9.84**	**14.91**
			**Opt-yes**	**12.29 ± 1.25**		
		**MinKE(Joule)**	**Opt-no**	**1.95 ± 0.20**	**1.52**	**2.39**
			**Opt-yes**	**2.01 ± 0.23**		
		**RangeKE(Joule)**	**Opt-no**	**10.43 ± 1.07**	**8.28**	**12.57**
			**Opt-yes**	**10.28 ± 1.05**		
		**MaxPE(Joule)**	**Opt-no**	**69.80 ± 3.37**	**62.88**	**76.71**
			**Opt-yes**	**70.00 ± 3.46**		
		**MinPE(Joule)**	**Opt-no**	**65.36 ± 3.05**	**59.06**	**71.66**
			**Opt-yes**	**65.63 ± 3.17**		
		**RangePE(Joule)**	**Opt-no**	**4.44 ± 0.37**	**3.73**	**5.15**
			**Opt-yes**	**4.37 ± 0.33**		
	**UKA**	**MaxKE(Joule)**	**Opt-no**	**10.83 ± 1.10**	**8.87**	**13.43**
			**Opt-yes**	**10.50 ± 0.97**		
		**MinKE(Joule)**	**Opt-no**	1.61 ± 0.19**	**1.24**	**2.09**
			**Opt-yes**	1.80 ± 0.20**		
		**RangeKE(Joule)**	**Opt-no**	**9.21 ± 0.93**	**7.58**	**11.39**
			**Opt-yes**	**8.70 ± 0.80**		
		**MaxPE(Joule)**	**Opt-no**	57.13 ± 2.12*	**52.21**	**61.65**
			**Opt-yes**	56.60 ± 2.13*		
		**MinPE(Joule)**	**Opt-no**	53.76 ± 1.93*	**49.24**	**57.91**
			**Opt-yes**	53.14 ± 1.97*		
		**RangePE(Joule)**	**Opt-no**	**3.37 ± 0.28**	**2.72**	**3.99**
			**Opt-yes**	**3.46 ± 0.29**		
**Lower leg**	**CG**	**MaxKE(Joule)**	**Opt-no**	**11.94 ± 0.44**	**11.07**	**12.82**
			**Opt-yes**	**11.92 ± 0.44**		
		**MinKE(Joule)**	**Opt-no**	**0.04 ± 0.00**	**0.04**	**0.05**
			**Opt-yes**	**0.04 ± 0.00**		
		**RangeKE(Joule)**	**Opt-no**	**11.90 ± 0.44**	**11.03**	**12.78**
			**Opt-yes**	**11.88 ± 0.44**		
		**MaxPE(Joule)**	**Opt-no**	**11.84 ± 0.31**	**11.21**	**12.48**
			**Opt-yes**	**11.86 ± 0.32**		
		**MinPE(Joule)**	**Opt-no**	**9.89 ± 0.26**	**9.37**	**10.42**
			**Opt-yes**	**9.86 ± 0.26**		
		**RangePE(Joule)**	**Opt-no**	**1.95 ± 0.05**	**1.83**	**2.08**
			**Opt-yes**	**2.00 ± 0.07**		
	**TKA**	**MaxKE(Joule)**	**Opt-no**	10.24 ± 0.97*	**8.18**	**12.30**
			**Opt-yes**	11.46 ± 1.06*		
		**MinKE(Joule)**	**Opt-no**	**0.05 ± 0.01**	**0.04**	**0.07**
			**Opt-yes**	**0.06 ± 0.01**		
		**RangeKE(Joule)**	**Opt-no**	10.19 ± 0.97*	**8.14**	**12.23**
			**Opt-yes**	11.40 ± 1.05*		
		**MaxPE(Joule)**	**Opt-no**	**14.14 ± 0.79**	**12.51**	**15.76**
			**Opt-yes**	**14.29 ± 0.81**		
		**MinPE(Joule)**	**Opt-no**	**11.65 ± 0.61**	**10.39**	**12.91**
			**Opt-yes**	**11.75 ± 0.64**		
		**RangePE(Joule)**	**Opt-no**	**2.49 ± 0.19**	**2.10**	**2.87**
			**Opt-yes**	**2.54 ± 0.19**		
	**UKA**	**MaxKE(Joule)**	**Opt-no**	**9.09 ± 1.00**	**7.16**	**11.02**
			**Opt-yes**	**9.30 ± 0.90**		
		**MinKE(Joule)**	**Opt-no**	0.04 ± 0.01***	**0.02**	**0.05**
			**Opt-yes**	0.06 ± 0.01***		
		**RangeKE(Joule)**	**Opt-no**	**9.05 ± 1.00**	**7.13**	**10.98**
			**Opt-yes**	**9.33 ± 0.90**		
		**MaxPE(Joule)**	**Opt-no**	11.59 ± 0.44*	**10.69**	**12.48**
			**Opt-yes**	11.36 ± 0.44*		
		**MinPE(Joule)**	**Opt-no**	9.63 ± 0.33*	**8.94**	**10.33**
			**Opt-yes**	9.49 ± 0.35*		
		**RangePE(Joule)**	**Opt-no**	**1.95 ± 0.13**	**1.72**	**2.18**
			**Opt-yes**	**1.87 ± 0.10**		
**Foot**	**CG**	**MaxKE(Joule)**	**Opt-no**	**12.44 ± 0.47**	**11.52**	**13.36**
			**Opt-yes**	**12.56 ± 0.45**		
		**MinKE(Joule)**	**Opt-no**	**0.00 ± 0.00**	**0.00**	**0.00**
			**Opt-yes**	**0.00 ± 0.00**		
		**RangeKE(Joule)**	**Opt-no**	**12.44 ± 0.47**	**11.52**	**13.36**
			**Opt-yes**	**12.56 ± 0.45**		
		**MaxPE(Joule)**	**Opt-no**	2.52 ± 0.07*	**2.38**	**2.67**
			**Opt-yes**	2.59 ± 0.08*		
		**MinPE(Joule)**	**Opt-no**	0.89 ± 0.02**	**0.85**	**0.93**
			**Opt-yes**	0.87 ± 0.02**		
		**RangePE(Joule)**	**Opt-no**	1.63 ± 0.05**	**1.52**	**1.74**
			**Opt-yes**	1.72 ± 0.06**		
	**TKA**	**MaxKE(Joule)**	**Opt-no**	**11.06 ± 0.83**	**8.34**	**12.24**
			**Opt-yes**	**26.29 ± 14.70**		
		**MinKE(Joule)**	**Opt-no**	0.63 ± 0.19**	**0.00**	**0.00**
			**Opt-yes**	0.71 ± 0.21**		
		**RangeKE(Joule)**	**Opt-no**	**10.43 ± 0.76**	**8.33**	**12.24**
			**Opt-yes**	**25.58 ± 14.72**		
		**MaxPE(Joule)**	**Opt-no**	**20.95 ± 4.72**	**2.73**	**3.59**
			**Opt-yes**	**20.94 ± 4.68**		
		**MinPE(Joule)**	**Opt-no**	**18.50 ± 4.56**	**1.09**	**1.46**
			**Opt-yes**	**18.41 ± 4.51**		
		**RangePE(Joule)**	**Opt-no**	**2.45 ± 0.23**	**1.62**	**2.14**
			**Opt-yes**	**2.53 ± 0.22**		
	**UKA**	**MaxKE(Joule)**	**Opt-no**	**9.08 ± 0.96**	**7.16**	**11.01**
			**Opt-yes**	**9.39 ± 0.93**		
		**MinKE(Joule)**	**Opt-no**	**0.00 ± 0.00**	**0.00**	**0.00**
			**Opt-yes**	**0.00 ± 0.00**		
		**RangeKE(Joule)**	**Opt-no**	**9.08 ± 0.96**	**7.16**	**11.01**
			**Opt-yes**	**9.39 ± 0.93**		
		**MaxPE(Joule)**	**Opt-no**	2.78 ± 0.13**	**2.55**	**3.01**
			**Opt-yes**	2.59 ± 0.10**		
		**MinPE(Joule)**	**Opt-no**	1.10 ± 0.40**	**1.02**	**1.19**
			**Opt-yes**	1.03 ± 0.04**		
		**RangePE(Joule)**	**Opt-no**	1.67 ± 0.09**	**1.52**	**1.83**
			**Opt-yes**	1.56 ± 0.07**		

Note: Opt-no: Leg without surgical operation; Opt-yes:Leg with surgical operation. KE is kinetic energy; PE is potential energy; CG is control group. *：*p*＜0.05; **: *p* ＜0.01; ***: *p* ＜0.001.

### 3.4 Potential energy parameters

#### 3.4.1 Control group versus TKA group

In the whole lower limb and its individual segments, both the operated and non-operated sides of the Control group showed significantly lower maximum and minimum values of PE compared to the TKA. In both the calf and foot, the PE ranges on the surgical side of the control group were lower than the PE ranges on both sides of the TKA group. The results are shown in Table 4, Table 5

#### 3.4.2 Control group versus UKA group

In the whole lower limb and its individual segments, compared to the Control group, the operated side of UKA had significantly higher max and min of PE. In the foot, the range of PE of the operated side in the UKA was significantly higher than that in the Control group. Detailed results can be found in Table 4, Table 5

#### 3.4.3 TKA group versus UKA group

There was no significant difference in PE between TKA and UKA as in Table 4, Table 5

#### 3.4.4 Non-operated side versus operated side

Both in the whole lower limb and its individual segments, the maximum and minimum values of PE on the operated side were significantly lower than those on the non-operated side in the UKA group. Furthermore, in the foot segment, the range of PE of the non-operated side in the UKA group was significantly higher than that of the operated side as in Table 4, Table 5

### 3.5 Recovery coefficient of lower limbs

**Fig. 7:**
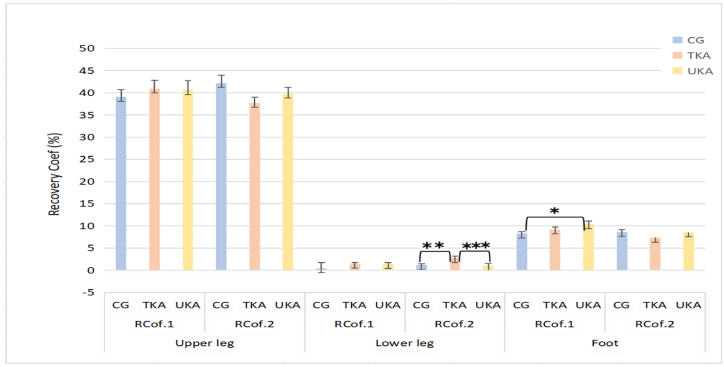
Comparison of recovery coefficients of the segments among the TKA, UKA, and Control groups. Note: a Covariates appearing in the model are evaluated at the following values: Age = 62.66， BMI = 27.98.b Adjustment for multiple comparisons: Bonferroni. c RCof.1 is energy recovery coefficient of leg without surgical operation; RCof. 2 is energy recovery coefficient of leg with surgical operation. CG is control group. *：*p*＜0.05; **: *p* ＜0.01; ***: *p* ＜0.001.

When comparing the lower leg segment, the Control group showed a significantly higher recovery coefficient on the operated side compared to the TKA group as in Table 4 and Fig. 7. Operated side of lower leg in TKA was significantly higher than UKA in Fig. 7.

**Fig. 8:**
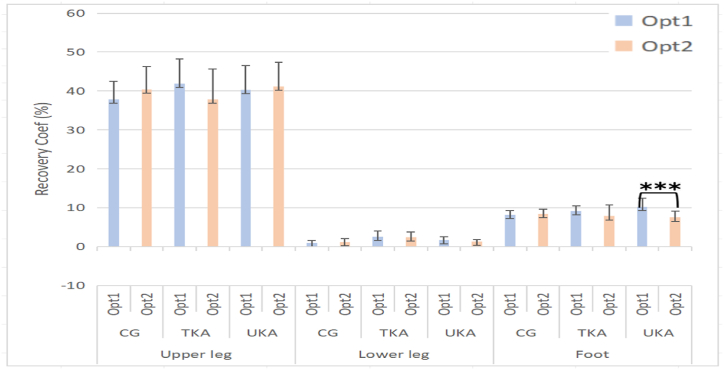
Comparison of recovery coefficients of the segments between operated side and non-operated side. Note: Opt-1: Leg without surgical operation; Opt-2: Leg with surgical operation. KE is kinetic energy; PE is potential energy; CG is control group. *：*p*＜0.05; **: *p* ＜0.01; ***: *p* ＜0.001.

When comparing the lower limb, the recovery coefficient on the non-operated side of UKA was significantly higher than on the operated side (Table 6). The recovery coefficient in the foot on the UKA operated side was significantly less than non-operated side as in Fig. 8.

### 3.6 Energy recovery in the center of pelvis

**Fig. 9:**
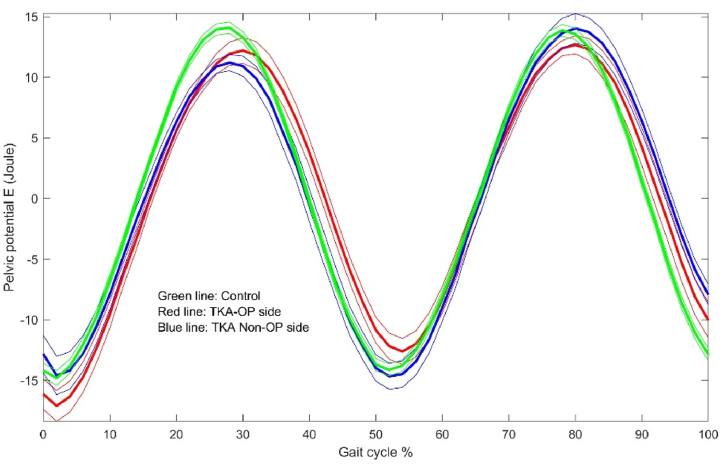
Comparison of potential energies between TKE op- and non-op sides and control groups in the pelvis.

**Fig. 10:**
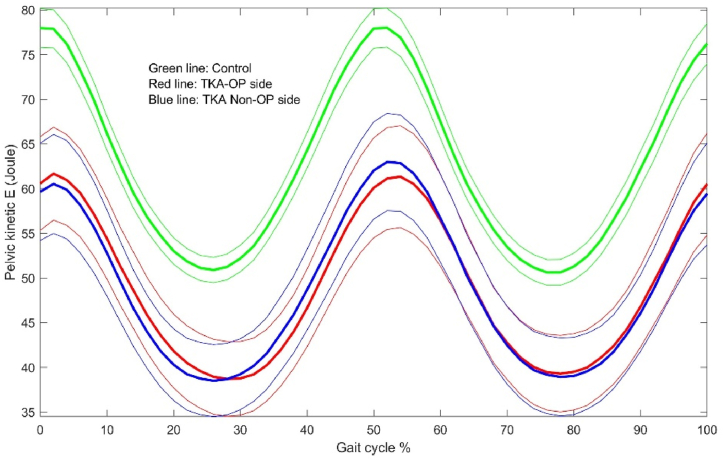
Comparison of kinetic energies between TKE op- and non-op sides and control groups in the pelvis.

The center of pelvis was thought as an approximate to the CoM of whole body [20]. In this study, the results showed that the recovery coefficient is higher in the control group than the TKA and UKA groups by roughly 12%–15%, while the TKA and UKA ones are not significantly different (Table 8). The energy dynamic changes at the center of pelvis are shown in Fig. 9, Fig. 10 between the control and TKA groups.

**Table 8:** Comparison of energy changes and recovery coefficients at the center of pelvis among groups.

Dependent Variable	Group	Mean ± SE	95% Confidence Interval		Notes
			Lower Bound	Upper Bound	
RangeKE	CG	32.4 ± 1.1	30.1	34.6	CG vs TKA**
	TKA	25.7 ± 1.3	23.1	28.2	CG vs UKA***
	UKA	25.9 ± 1.2	23.5	28.3	TKA vs UKA
RangePE	CG	32.8 ± 1.2	30.4	35.2	CG vs TKA
	TKA	28.8 ± 1.4	26.1	31.5	CG vs UKA
	UKA	29.8 ± 1.3	27.2	32.3	TKA vs UKA
RangeTKE	CG	32.4 ± 1.1	30.1	34.6	CG vs TKA**
	TKA	25.7 ± 1.3	23.1	28.2	CG vs UKA***
	UKA	25.9 ± 1.2	23.5	28.3	TKA vs UKA
RecoveryCof	CG	80.1 ± 1.2	77.7	82.4	CG vs TKA***
	TKA	70.4 ± 1.4	67.7	73.1	CG vs UKA***
	UKA	72.8 ± 1.3	70.3	75.3	TKA vs UKA

Notes: a. Covariates appearing in the model are evaluated at the following values: BMI = 27.9927.

b. CG is control group. *：p＜0.05; **: p ＜0.01; ***: p ＜0.001.

## 4 Discussion

In this study, the difference in lower limb energy during walking between operated side and non-operated side, and among Control group, TKA and UKA were compared. In this study, we adhere to strict inclusion criteria to ensure that participants align with the research objectives. The implementation of these measures is aimed at minimizing the potential interference of participants' medical histories with the study results. Although this study found significant differences in body weight and age among the three groups, these differences did not impact the mechanical energy exchange between the groups after we accounted for BMI and age as covariates and treated group and gender as fixed factors in our statistical analysis (as shown in Table 4, Table 5, Table 6, Table 7). Regarding gender distribution, our study revealed no significant differences in gender among the three groups. We conducted statistical analyses using BMI and age as covariates and considering group and gender as fixed factors, and these analyses indicated that these factors had no effect on the mechanical energy exchange among the three groups. The study results indicate that in our research, gender's influence was effectively controlled, allowing for a comprehensive exploration of mechanical energy exchange.

### 4.1 Gait parameters

In this study, patients after UKA demonstrated similar gait parameters to those after TKA. However, interestingly, despite the similarity in gait parameters, the average walking speed for TKA patients was 0.94 m/s, whereas for UKA patients, it was 1.09 m/s, both of which were much slower than speeds reported in previous studies [[21], [22], [23]]. We speculate that this discrepancy may be attributed to age, as age has been associated with decreased walking speed [24,25]. In our study, the walking speeds of both UKA and TKA patients were similar to those of healthy adults in the same age range, as reported by Nishizawa et al. [26]. Regarding spatiotemporal gait parameters, our study showed no significant differences between the TKA and UKA groups (see Table 3), suggesting that their clinical impact may not be substantial.

Furthermore, we found that TKA patients had significantly slower average walking speed compared to the control group. In terms of cadence, both the operated and non-operated sides of TKA and UKA were lower than the control group, indicating that TKA and UKA patients might have had insufficient recovery time, and even the cadence of the unaffected side in both UKA and TKA patients was lower than that of the control group. This may be because postoperative patients often use their unaffected leg as a supporting leg to protect the operated side, leading to lower cadence.

### 4.2 Kinetic energy

Mechanical energy expenditure largely reflects an individual's functional performance and capacity [27]. Kinetic energy is closely related to mass and speed. Some studies have shown that speed affects gait parameters [28,29], but this study found no significant variability in gait parameters such as stride length, cadence and walking speed between the TKA and UKA. So in this study, there was little difference in kinetic energy between two groups, and no significant difference occurred between TKA and UKA. The differences were mainly manifested in the surgical and non-operative sides of the patients, and between the Control group and TKA.

In this study, we found that in the lower leg segment, the maximum kinetic energy and kinetic energy range of the operated side in the TKA group were higher than those in the control group. However, at the same time, the walking speed of the TKA group was significantly slower than that of the control group. This suggests that, compared to healthy individuals, TKA patients need to expend more kinetic energy to complete their gait. Wang et al.'s study [19] found that comfortable walking speed is associated with optimal mechanical performance for energy exchange and recovery. Therefore, we speculate that the reason TKA patients do not achieve better walking speed postoperatively may be because their postoperative recovery has not reached the optimal mechanical performance for energy exchange and recovery.

In this study, these findings indicate a notable increase in kinetic energy on the operated side relative to the non-operated side in patients after TKA and UKA surgeries. The reason for this may be related to the volunteer's post-operated walking pattern. One study showed that TKA patients still walked 18 percent slower than normal patients one year after surgery [30]. Another study showed that knee replacement patients' walking speed did not improve significantly between 0.5 and 5 months after surgery, but then showed high efficiency for 6–60 months, with a small decrease beginning at 13 months [29,31]. The reason for this may be related to the walking pattern of subjects.

### 4.3 Potential energy

Potential energy is closely related to the calculation of mass and height. In this study, we used the ground as the reference point for height. Our research findings confirmed that the center of mass height achieved during gait is lower in the control group than in post-TKA and post-UKA patients. Mechanical efficiency is defined as the energy consumed to perform a certain amount of external work. Some studies found that a comfortable walking speed is associated with higher energy exchange and recovery [10]. The higher potential energy in postoperative patients may be attributed to the need for adopting compensatory gait patterns to reduce fatigue. These findings reveal potential adaptive adjustments in gait control among postoperative patients. Levinger et al. [32] reported that after TKA, the biomechanics of the ankle joint also changed, and the torques of dorsiflexion and plantarflexion increased significantly, which was a compensation for the insufficient ability of the knee joint to provide sufficient walking power.

This study indicated that the lower limbs on the non-operated side are more active than those on the operated side when walking on the vertical plane, which may be a result of reduced maximum potential energy due to hip flexion contracture after UKA surgery. In contrast to TKA, knee flexion and extension patterns are maintained well in most UKA patients during walking cycles [33]. This is also consistent with the results obtained in our study. This may be that the knee or hip joint in TKA is more active in the vertical plane than in the UKA group, or it may be influenced by the weight difference between the two groups. Chassin et al. [34] reported that there was more knee flexion in the UKA group than in the TKA group during walking, which may be related to the results of this study.

### 4.4 Recovery coefficient

The concept of Recover was proposed a long time ago. Wang et al. [19] showed that among different walking modes, the way of subjective comfortable walking had the highest energy recovery efficiency and was also considered to be the walking state with the least effort. In this study, the main analysis is the conversion efficiency of kinetic energy and potential energy of lower limbs when walking.

In terms of energy exchange in the lower limb segments, our study found that in the UKA group, the non-operated side of the foot segment exhibited significantly higher energy exchange compared to the operated side. Additionally, when comparing the same operated side, the recovery coefficient of the TKA group's lower leg was significantly higher than that of the UKA and control groups. These results suggest that TKA surgery is more favorable for recovery in the lower leg, while UKA surgery is more beneficial for recovery in the thigh, possibly due to different surgical approaches.

Furthermore, based on all the statistical data, we found that the upper limbs had the highest energy conversion rate during walking (approximately 40%), followed by the feet (approximately 10%) and the lower legs (1–3%). In other words, the upper limbs were more efficient in energy conversion compared to the lower legs and feet, which aligns with the principle of a pendulum. To our knowledge, these findings are the first to be discovered in gait analysis. Therefore, these results will undoubtedly contribute to our understanding of the gait in patients after TKA or UKA surgeries and support our clinical rehabilitation practices.

### 4.5 Limitation

There are some limitations in this study, the most important of which is the lack of relevant preoperative walking data. However, it was observed that the gait parameters at 4 months post-surgery were better predictors of abnormal gait parameters at 1-year post-surgery than preoperative gait parameters [35]. Due to the impact of COVID-19, the data for the three groups in our study had to be directly extracted from a database. However, it's important to note that all the data in the database originate from the local region, which means we couldn't increase the sample size or achieve equal group size. Due to the small sample size, the applicability of the study's results is limited to the specific characteristics of the participants and may not necessarily represent a broader population. As a small sample size, this study was defined as a preliminary study as in the title. Therefore, we hope that future research will increase the sample size and employ more comprehensive data collection methods to gain a more comprehensive understanding and evaluation of the gait performance and recovery coefficients of postoperative patients. In addition, the ages from different groups were different, which could affect the energy changes during gait. However, the energy recovery coefficients are dimensionless and thus comparable among groups.

### 4.6 Clinical relevance

As there is no reseach on analyses of lower limb energy recovery coefficients in patients after UKA and TKA surgery, the results of this study can serve as a supplement to this new field. The energy recovery coefficient values have clinical potential in applications. In terms of the heavy calculations, this project made a specific computer program (using Matlab®) which could be combined into a standard clinical analysis package, e.g. Vicon Nexus®. Therefore, clinicians could run it quickly and routinely.

In practical application, all calculations on energy parameters would be carried out by software, taking a couple of seconds, and thus what clinicians need to do is to explain the results, e.g. which group is higher or lower than the control group in the parameters. This information could provide a specific aspect of gait for the patients with movement disordered.

## 5 Conclusion

In this study, the comparisons among the control group, TKA group, and UKA group primarily focused on potential energy. Both the TKA and UKA groups exhibited significantly higher maximum and minimum potential energy in the entire lower limb and individual segments compared to the control group. Although there were no statistically significant differences in potential energy between the TKA and UKA groups, except for the foot segment on the operated side, the TKA group showed larger potential energy variations in all other segments compared to the UKA group. Regarding the comparison of recovery coefficients, based on all the statistical data, the upper legs had the highest energy conversion rate during walking (approximately 40%), followed by the feet (approximately 10%) and the lower legs (1–3%). However, the energy recovery coefficients at the centre of pelvis were significantly higher in the control group than the TKA and UKA groups by roughly 12%–15%.

**Lab inf:** the Motion & Gait Analysis Laboratory at the Tayside Orthopaedics and Rehabilitation Technology Centre (TORT), School of Medicine, Ninewells Hospital, Dundee, Scotland.

## Funding information

The 10.13039/100008890University of Dundee the Library's Institutional Open Access Fund.

## Ethical note

A waiver of ethical approval. As this study extracted the data from the existing database without contacting participants, the school ethical committee granted the waiver of ethical application. The lab is on the base of a hospital where there is a general ethical approval for data collection and subjects had signed written consent forms when they attended the original data collection. When data was collected previously, ethical approval was granted by University of Dundee Research Ethics Committee (UREC 15183) and the Cambridge South Research Ethics Committee (16/EE/0021).

## Data availability statement

Data will be made available on request.

## CRediT authorship contribution statement

**Haifei Zhou:** Investigation, Formal analysis, Data curation. **Yuying Zhang:** Writing – original draft, Visualization, Investigation, Formal analysis. **Archit Agarwal:** Data curation. **Graham Arnold:** Resources, Project administration, Data curation. **Weijie Wang:** Writing – review & editing, Validation, Supervision, Software, Methodology, Investigation, Funding acquisition, Formal analysis, Conceptualization.

## Declaration of competing interest

The authors declare that they have no known competing financial interests or personal relationships that could have appeared to influence the work reported in this paper.
